# Glycosylation modification patterns reveal distinct tumor metabolism and immune microenvironment landscape in lower-grade gliomas

**DOI:** 10.3389/fcell.2022.886989

**Published:** 2022-08-25

**Authors:** Guihua Tang, Liming Tan, Hao Yuan, Wen Yin

**Affiliations:** ^1^ Department of Clinical Laboratory, Hunan Provincial People’s Hospital, The First Affiliated Hospital of Hunan Normal University, The College of Clinical Medicine of Human Normal University, Changsha, China; ^2^ Department of Neurosurgery, National Clinical Research Center for Geriatric Disorders, Xiangya Hospital of Central South University, Changsha, China

**Keywords:** glycosylation, lower-grade gliomas, prognosis, tumor metabolism, immune microenvironment landscape

## Abstract

Glycosylation alterations, a key driver throughout tumorigenesis and tumor progression, could regulate the microenvironment and immune response as well as lead to harmful metabolism and cell signaling. In this study, we first comprehensively evaluated the glycosylation modification patterns of LGGs based on glycosyltransferase family genes and systematically integrated these modification patterns with tumor metabolism and immune microenvironment characteristics. Glycosylation score was also developed to quantify glycosylation modification patterns of individuals. As a result, two glycosylation modification patterns were identified, with distinct prognosis, metabolism, and immune microenvironment features. The glycosylation subtype A and cluster A were characterized by higher carbohydrates and amino acid metabolism activity, higher levels of infiltrating cells, and poor prognosis, whereas an opposite modification pattern was observed in glycosylation subtype B and cluster B. In addition, a high glycosylation score is closer to a microenvironment characterized by chronic inflammation, immunosuppression, and tumor promotion. Following analysis and validation, the glycosylation score was a reliable and independent prognostic index. More importantly, the glycosylation score influenced the response to immunotherapy, chemotherapy, or targeted therapy, which provided a novel insight into promoting personalized therapy in the future and may contribute to developing novel therapeutic drugs or exploring promising drug combination therapy strategies.

## Introduction

Diffuse gliomas, a heterogeneous group of the supporting glial cell neoplasms, are the most prevalent primary malignancies of the central nervous system (CNS). The World Health Organization (WHO) classified gliomas into grades I–IV (Yu H. et al., 2021). Grades II and III gliomas are often defined as lower-grade gliomas (LGGs) because these tumors represent a completely different entity from glioblastoma (grade IV). Up to 80% of LGGs harbor isocitrate dehydrogenase (IDH) mutations, but only approximately 5% of glioblastomas are IDH-mutant (Youssef and Miller, 2020). More importantly, this molecular finding conveys a relatively optimistic and favorable prognosis (Schiff et al., 2019). Complete surgical resection followed by chemoradiotherapy is the currently favored therapy for LGGs (Schiff et al., 2019; Yu H. et al., 2021). However, LGGs cannot be cured completely, and more than 70% of patients will inevitably experience tumor recurrence or malignant progression within 10 years (Gittleman et al., 2020), owing to its high invasiveness and aggressiveness. Thus, reducing tumor recurrence and delaying tumor progression are the most issues and challenges. Even after active efforts of applying multiple treatment modalities, however, the malignant behaviors and clinical outcomes of these tumors vary greatly from person to person (Aoki et al., 2018; Berzero et al., 2021). Due to the high heterogeneity of LGGs, it was insufficient for the existing molecular patterns of LGGs to fully explain clinical outcomes and predict prognosis. Therefore, more comprehensive research to better understand potential molecular mechanisms in LGGs oncogenesis and progression is needed, which will contribute to better patient stratification and further making clinical decisions to improve patient management.

Glycosylation, a recognized hallmark of cancer(Thomas et al., 2021), is defined as an enzymatic process that could accelerate the production of glycosidic linkages between saccharides and other saccharides, lipids, or proteins (Pinho and Reis, 2015). Emerging evidence demonstrated that altered glycans serve as a key driver throughout tumorigenesis and tumor progression; hence, changes in cellular glycosylation, as a key component of malignant progression, have recently attracted more and more attention (Stowell et al., 2015). Glycosylation is involved in almost all important biological processes, such as protein quality control, protein clearance, intracellular trafficking, cell–cell interaction, cell–matrix adhesion, and various signal transduction cascades (Thomas et al., 2021). In addition, abnormal and modified glycosylation alterations caused by cellular and metabolic changes will not only significantly affect the overall charge and conformation of glycoproteins, thereby changing its biological activity and directly affecting tissue cell growth and survival (Stowell et al., 2015). Also, these changes of glycosylation also cause abnormal expression of membrane-localized glycans, which will trigger cellular malignant transformation and mediate cancer cell proliferation, survival, and metastasis (Marsico et al., 2018; Chandler et al., 2019; Thomas et al., 2021). Accumulating findings suggest that abnormal glycan profiles could regulate the microenvironment and immune response (Mereiter et al., 2019) and even hinder an effective immune response (Polmear and Good-Jacobson, 2021). In addition, the metabolic state (Campbell and Wellen, 2018) or metabolic reprogramming (Carvalho-Cruz et al., 2018) of the tumor cell can lead to aberrant glycosylation intra- and extra-cellular. On the contrary, aberrant glycosylation can also lead to harmful metabolism and cell signaling. However, these mechanisms are not fully understood. Therefore, evaluating the association between glycosylation modification patterns and immune landscape, along with metabolism alterations will contribute to strengthening our understanding of the vital role of glycosylation in tumor biology, and is expected to provide valuable strategies for improving personalized management of patients.

In the present study, we integrated the transcriptomic information of LGGs samples to identify the glycosylation patterns associated with distinct prognosis and immune and metabolic characteristics. Moreover, we first proposed a scoring system to quantify the glycosylation patterns of individual LGGs patients, and it can provide clinical decision-makers with a novel perspective to better stratify patients, predict treatment response, and improve individualized treatment strategies.

## Materials and methods

### Data source and preprocessing

The overview of our study design is shown in Supplementary Figure S1. The LGGs gene expression data and matched clinical annotations were retrieved from TCGA and Chinese Glioma Genome Atlas (CGGA) databases. Only patients with survival time greater than 30 days were involved in this study. In total, four cohorts were enrolled: TCGA-LGG (481 samples), CGGA-mRNAseq_693 (420 samples), CGGA-mRNAseq_325 (170 samples), and CGGA-mRNA-array_301 (159 samples). The TCGA-LGG cohort data by log2(x+1) transformed were downloaded from the UCSC Xena website (https://xena.ucsc.edu/), and the other three cohorts were downloaded from the CGGA official website (http://www.cgga.org.cn/index.jsp). At the same time, the mRNA sequencing (mRNA-seq) data in CGGA was also log2(x+1) normalized.

### Glycosylation-based consensus clustering analysis

We collected the genes of the main glycosyltransferase families involved in human species-specific glycosylation mechanisms (Mohamed Abd-El-Halim et al., 2021), including 221 genes retrieved from the GlycoGene Database (GGDB, http://riodb.ibase.aist.go.jp/rcmg/ggdb) and 213 genes extracted from the Hugo Gene Nomenclature Committee (HGNC, https://www.genenames.org/data/genegroup/#!/group/424). Eventually, a total of 143 overlap genes were selected in our analysis. Consensus clustering (Zhang et al., 2020) was performed to identify distinct glycosylation subtypes according to the expression of these 143 genes in the TCGA cohort. The ConsensuClusterPlus package was used and 1000 times repetitions were conducted to ensure the stability of clustering results. In addition, we applied the T-distributed stochastic neighbor embedding (t-SNE) to verify the reliability of subtype assignments based on the expression of the aforementioned genes (Bai et al., 2021).

### Gene set variation analysis

To explore the variations in biological processes between glycosylation modification patterns, we selected the GSVA enrichment analysis to accomplish this purpose by using the “GSVA” package. GSVA is usually applied to estimate the variation of pathway and biological process activity in a nonparametric and unsupervised manner (Hänzelmann et al., 2013). The gene sets of “c2.cp.kegg.v7.4.symbols”, GOBP, and metabolism-associated pathway signatures derived from KEGG and REACTOME were both downloaded from the molecular signature database (MsigDB, http://www.gsea-msigdb.org/gsea/msigdb/search.jsp). Adjusted *p* < 0.05 was considered statistically significant.

### Estimation of tumor microenvironment characteristics

The single sample gene set enrichment analysis (ssGSEA) was applied to estimate the enrichment scores of 13 immune functions and 16 immune cells. The scores were normalized to specific distribution from 0 to 1 and could quantify the relative abundance of estimated items. The marker gene sets were curated from a current study (Bindea et al., 2013), and the “GSVA” package was introduced to conduct the aforementioned analysis. Moreover, to comprehensively estimate the infiltration level of immune cells and stromal cells in the TME, Immune Cell Abundance Identifier (ImmuCellAI) (Miao et al., 2020), Tumor Immune Estimation Resource (TIMER) (Yu L. et al., 2021), and microenvironment cell population counter (MCP-counter) (Ye et al., 2021) algorithms were also utilized in our study. Among them, the former is calculated in the web tool ImmuCellAI (http://bioinfo.life.hust.edu.cn/ImmuCellAI#!/), while the latter two algorithms are implemented on the Sangerbox platform (http://vip.sangerbox.com/home.html).

### Identification of glycosylation-related hub genes

To identify glycosylation-related genes (GRGs), we performed differential expression analysis between glycosylation distinct subtypes. The “limma” package was used to determine differentially expressed genes (DEGs), and the significance filtering criteria were set as | log2 (fold change) | > 1 as well as adjusted *p* value less than 0.0001. In addition, to ensure the reliability of GRGs, weighted gene co-expression network analysis (WGCNA) was also introduced to establish co-expressed gene modules strongly related to glycosylation. Based on the expression of 8619 prognostic genes (univariate Cox analysis *p* < 0.01), a gene co-expression network was established by using the “WGCNA” R package (Langfelder and Horvath, 2008). The Pearson correlation was calculated according to the expression of these filtered genes, and an optimal soft-thresholding power *β* = 7 was selected to build an unsigned weighted adjacency matrix followed by a topological overlap matrix (TOM) conversion. Finally, the average linkage hierarchical was clustered with the parameter height = 0.25, and the criterion for gene module identification was set as a cutting height of 0.9 along with the module genes a minimum number of 20. The modules of | correlation coefficient | > 0.5 were considered as closely related to glycosylation and selected for a subsequent analysis. The intersections of DEGs and module genes screened by WGCNA were defined as GRGs.

### Generation and validation of glycosylation gene signature and glycosylation score

To quantify the glycosylation modification patterns of individual patients, we developed a glycosylation scoring scheme to assess the glycosylation modification pattern of individuals with LGGs, which was glycosylation gene signature and the glycosylation score. Primarily, we divided the GRGs into two groups based on their hazard ratio (HR) of the univariate Cox regression model. Genes with HR > 1 and those with HR < 1 were defined as the glycosylation gene signatures A and B, respectively. Next, we conducted GSVA for dimension reduction of the signatures A and B, the GSVA value of which were separately termed as value A and value B. Finally, both value A and value B were selected to calculate the glycosylation score as follows: glycosylation score = value A- value B. Patients with LGGs were classified into high and low glycosylation score groups based on the median cutoff value, and scores between the glycosylation subtypes or clusters were assessed by the Wilcoxon test.

Moreover, the prognostic value of the glycosylation scores was validated in three cohorts of the CGGA database by using the same signatures and the cutoff value.

### Correlation between the glycosylation score and other related biological processes

To explore the association between the glycosylation score and some related biological pathways. The gene sets related to stromal activation were extracted from the supplementary material of a current study (Mariathasan et al., 2018), including epithelial–mesenchymal transition (EMT) signatures (EMT1, EMT2, and EMT3) and pan-fibroblast TGFb response signature (Pan-FTBRS). Moreover, immune checkpoint- and immune activation–related genes were retrieved from the publication of Zhang et al. (2020). In addition, immunosuppressive factors and immunosuppressive cell recruitment factors were obtained from another study (Su et al., 2019). The differences in biological process between distinct glycosylation score groups were assessed *via* the Wilcoxon test.

### Small molecule drugs screening and drug sensitivity prediction

Differentially expressed genes (DEGs) between the high and low glycosylation score groups were set as adjusted *p*-value < 0.001 and | log2 (fold change) | > 1, which were determined by using the “limma” package. The DEGs were visualized into volcano plots and submitted to perform Gene Ontology (GO) and Kyoto Encyclopedia of Genes and Genomes (KEGG) enrichment analyses by the “clusterProfiler” package. Furthermore, both of the top 500 up- and downregulated genes were uploaded into the Connectivity Map 02 (CMap, https://portals.broadinstitute.org/cmap/) database, and a CMap mode-of-action (MoA) analysis was applied to discover possible small molecular drugs for LGGs and mechanisms of action.

We evaluate the predictive capacity of glycosylation score in responding to chemotherapeutic agents and immunotherapy. The 50% inhibiting concentration (IC50) value of 138 drugs of each patient was quantified using the pRRophetic algorithm (Tan et al., 2021), and the differences between high and low glycosylation score subgroups compared by using the Wilcoxon test. In addition, the response to immunotherapy was predicted by using immunophenoscore (IPS) (Gui et al., 2021; Wu et al., 2021), which was obtained without bias based on determining components of immunogenicity including effector cells, immunosuppressor cells, major histocompatibility complex (MHC) molecules, and immune modulators. The statistical difference of the IPS value was tested by the Wilcoxon test.

### Statistical analysis

R software (version 4.1.0) was applied to conduct statistical analysis. We adopted the Kaplan–Meier (K-M) analysis and log-rank tests to perform the survival analysis with the “survival” R package. The prognostic value of glycosylation score was evaluated by a multivariate Cox regression model and a time-dependent receiver operating characteristic (ROC) analysis using R packages “survival’ and “timeROC”. Comparisons between glycosylation subgroups, clusters, and score groups were presented *via* the Wilcoxon test.

## Results

### Glycosylation-related molecular subtypes with distinct survival, metabolism, and immune microenvironment features in LGGs

**FIGURE 1:**
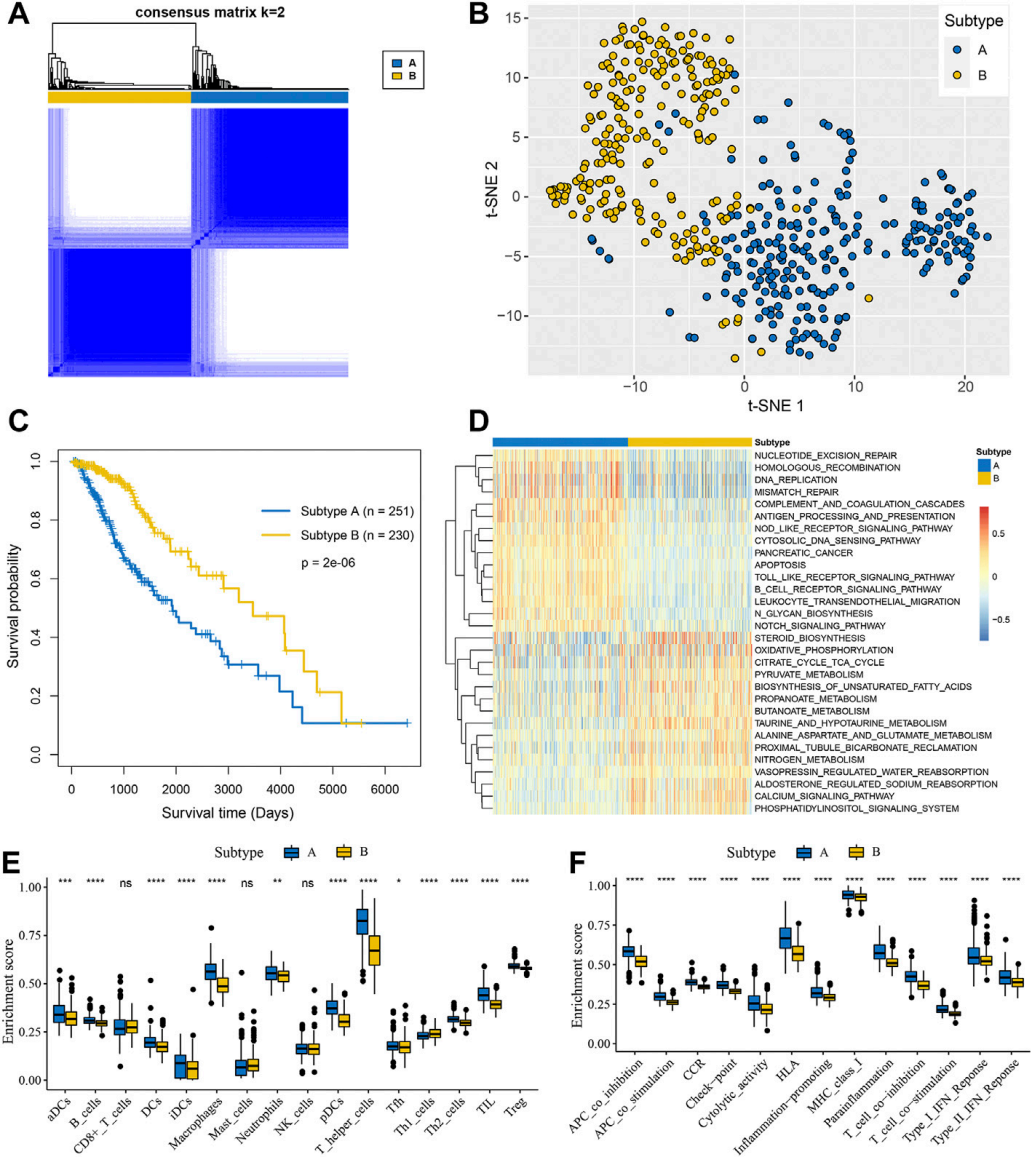
Glycosylation-related molecular subtypes with distinct survival and immune microenvironment features in LGGs. **(A)** Consensus clustering matrix of 481 samples from the TCGA dataset for *k* = 2. **(B)** t-SNE of the expression profiles of glycosyltransferase family genes in the TCGA cohort confirmed the subtypes. **(C)** K-M analysis for the subtypes of LGGs patients. **(D)** GSVA enrichment analysis displayed the differences of KEGG pathways between the two subtypes. Red represented higher pathway activity and blue represented lower pathway activity. The boxplots visualized the ssGSEA enrichment scores of immune infiltrating cells **(E)** and immune functions **(F)** between the subtypes. *p < 0.05; **p < 0.01; ***p < 0.001; ****p < 0.0001; ns, not significant.

Based on the expression of 143 genes involved in glycosyltransferase families, 481 patients from the TCGA database were obviously divided into two groups when *k* = 2 (Figure 1A) by the consensus clustering analysis, which was termed as glycosylation subtype A and subtype B, respectively. The t-SNE algorithm confirmed that glycosylation subtype assignments are reliable and can be completely distinguished (Figure 1B). The survival analysis revealed that patients of subtype A showed a significantly shortened survival time compared with patients of subtype B (Figure 1C, log-rank test).

**FIGURE 2:**
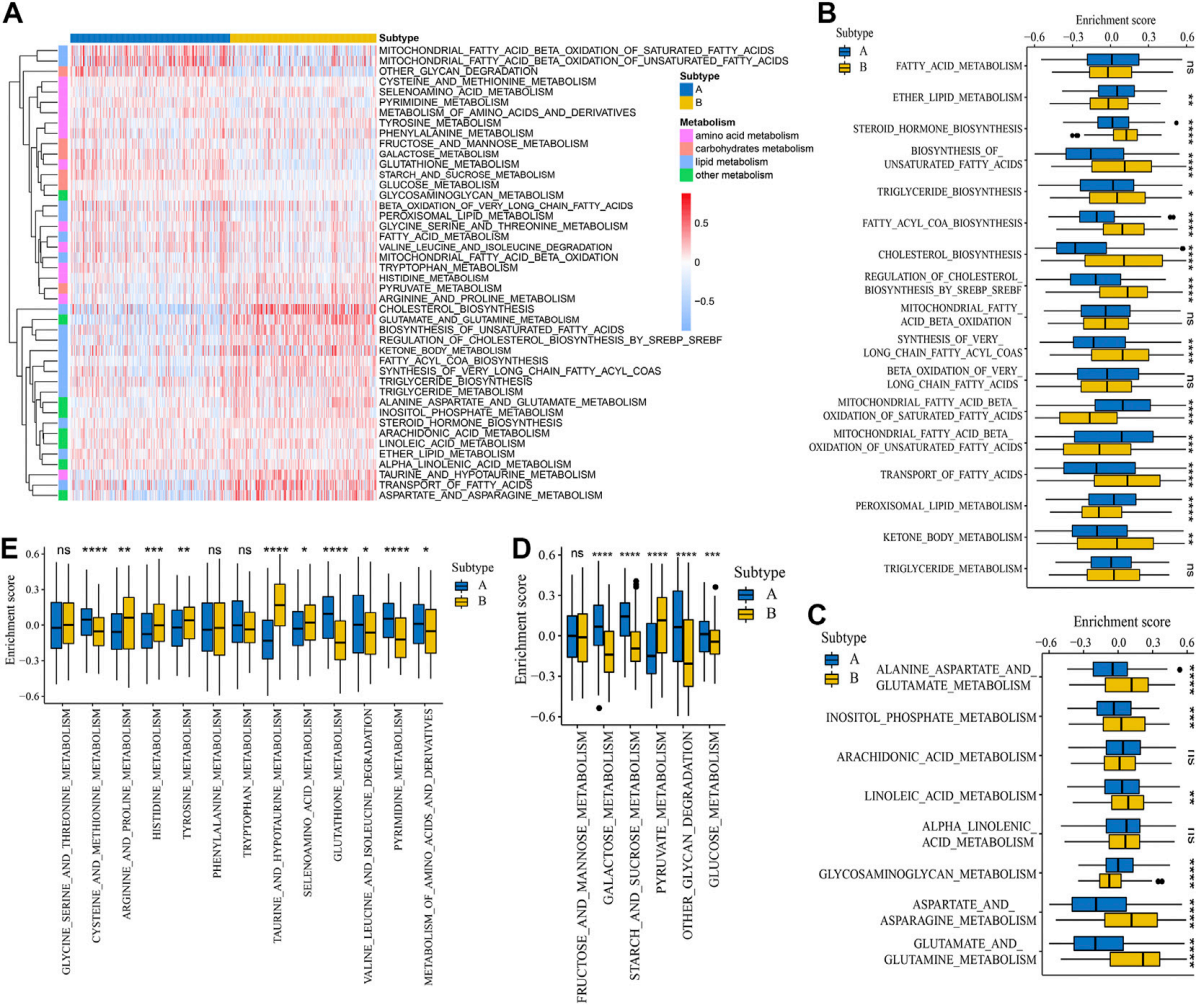
Metabolism features of the glycosylation subtypes. **(A)** Heat map depicted the activation states of metabolism-associated process between subtypes based on GSVA enrichment scores. Red represents activated processes and blue represents inhibited processes. Differences of GSVA enrichment scores of the metabolism-associated process including lipid metabolism **(B)**, other metabolism **(C)**, carbohydrates metabolism **(D)**, and amino acid metabolism **(E)** between the subtypes. *p < 0.05; **p < 0.01; ***p < 0.001; ****p < 0.0001; ns, not significant.

To explore the biological molecular changes between distinct glycosylation subtypes, the KEGG gene set was used to performed GSVA enrichment analysis. As shown in Figure 1D, subtype A presented enrichment pathways prominently related to carcinogenic activation and immune regulation pathways such as nucleotide excision repair, homologous recombination, antigen processing and presentation, and NOD-like receptor signaling pathway, while subtype B is markedly enriched in metabolism-related processes, including steroid biosynthesis, oxidative phosphorylation, citrate cycle TCA cycle, and so on. Next, we further compare the metabolism and immune microenvironment features affected by glycosylation modification patterns between the subtypes. The results of ssGSEA showed a significant up-regulation in immune infiltrating cells (Figure 1E) and immune function (Figure 1F) of subtype A. Furthermore, the GSVA results revealed significant differences in metabolic processes (Figure 2A), with subtype B mainly active in lipid metabolism (Figure 2B) and other metabolism (Figure 2C), while subtype A was mainly active in carbohydrates metabolism (Figure 2D) and amino acid metabolism (Figure 2E).

### Survival, metabolism, and immune microenvironment characteristics in glycosylation clusters for LGGs

**FIGURE 3:**
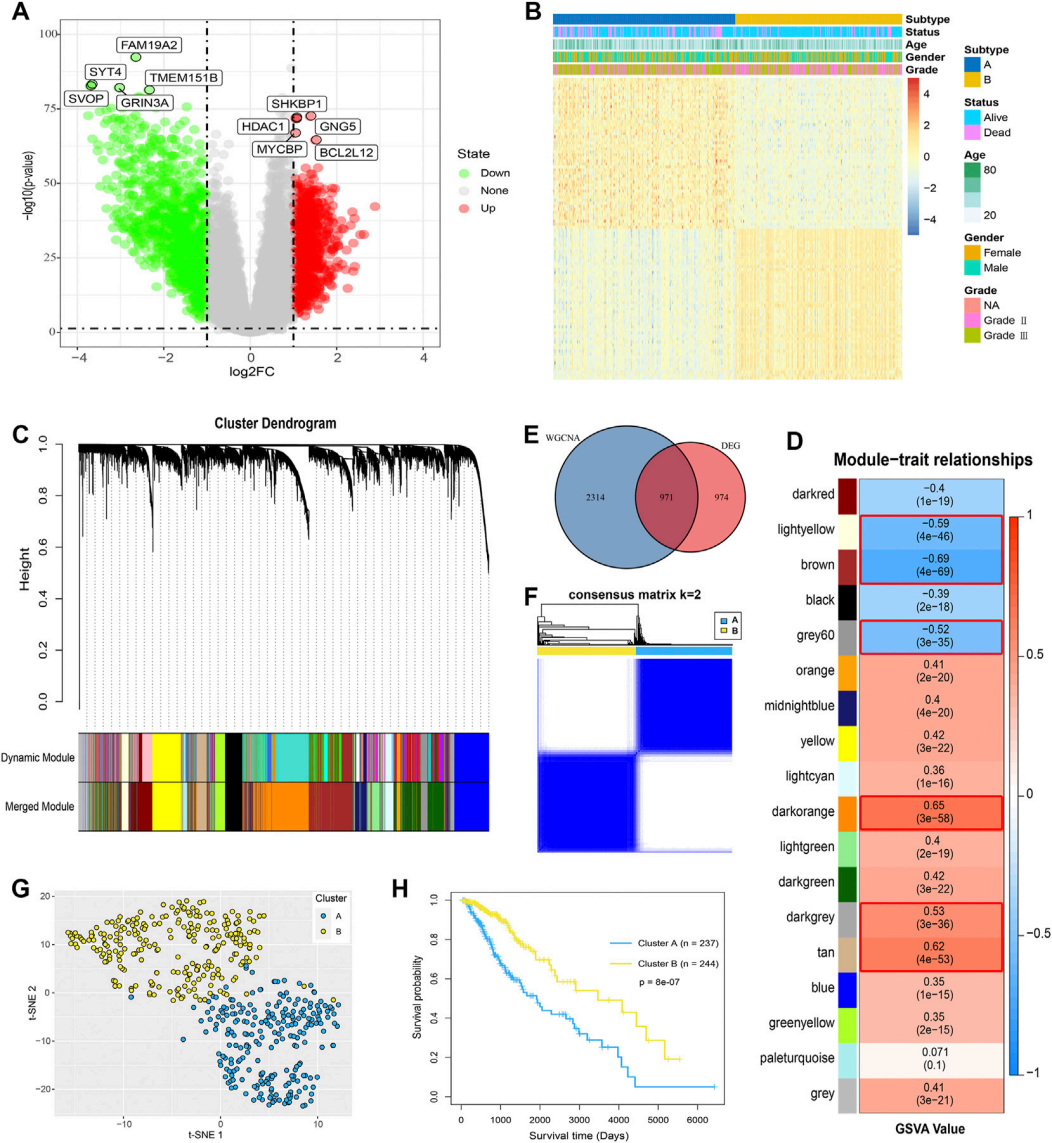
Identification of GRGs and glycosylation clusters. **(A)** Volcano map showed the DEGs between the glycosylation subtypes. Red represented up-regulated genes and green represented down-regulated genes. **(B)** Heat map showed clinical features and top 100 DEGs between glycosylation subtypes. **(C)** Gene co-expression network and gene modules were identified by WGCNA. **(D)** In total, 18 gene modules were recognized, and six modules marked with red frames, with | correlation coefficient | > 0.5, were considered as closely related to glycosylation. **(E)** In total, 971 overlapping genes were determined by DEGs and WGCNA were defined as GRGs. **(F)** Consensus clustering matrix for *k* = 2 and **(G)** t-SNE identified glycosylation clusters, which were both based on the expression profiles of GRGs in the TCGA cohort. **(H)** K-M analysis for the clusters of LGGs patients.

**FIGURE 4:**
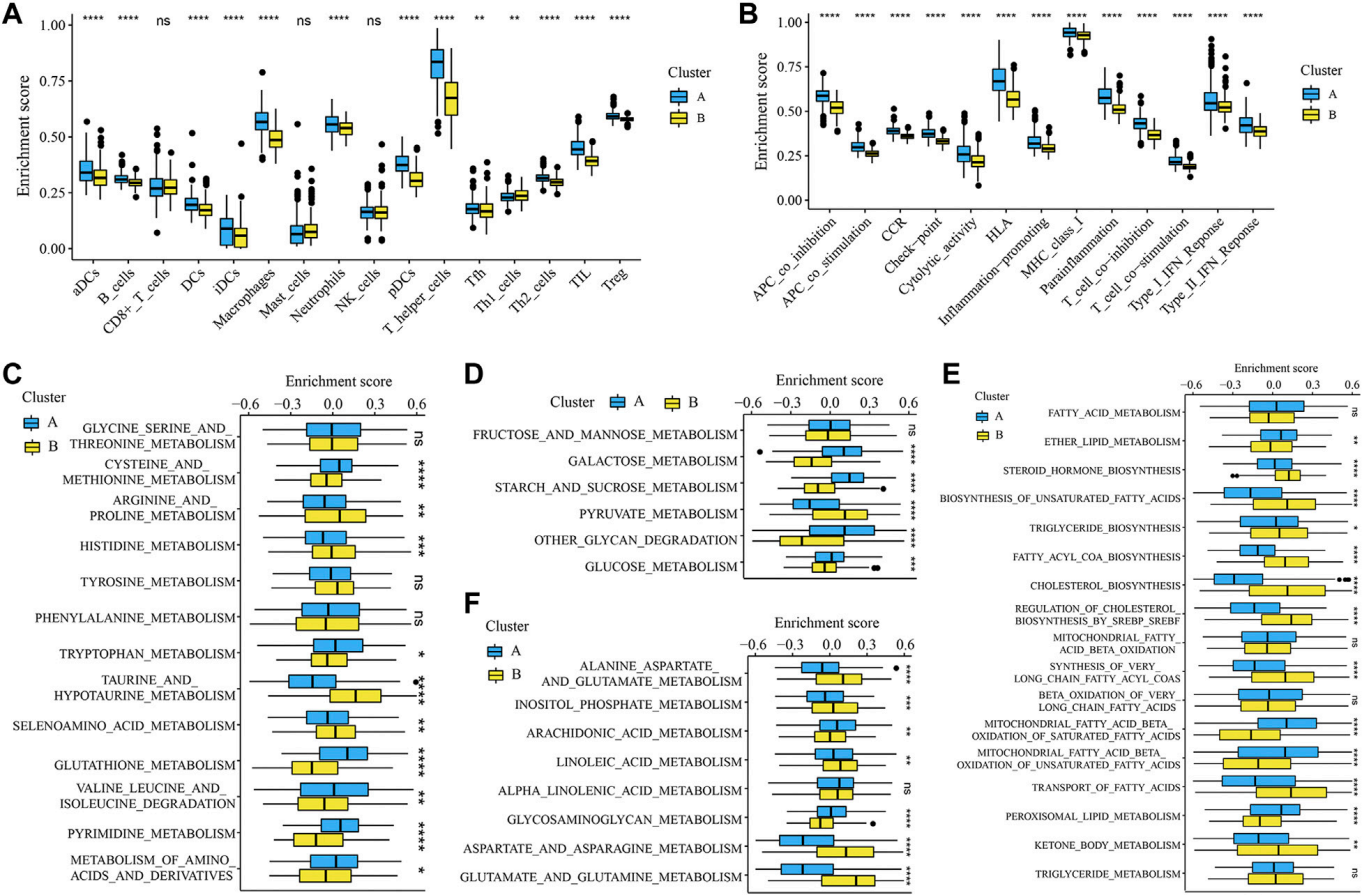
Immune microenvironment and metabolism features of the glycosylation clusters. *p < 0.05; **p < 0.01; ***p < 0.001; ****p < 0.0001; ns, not significant. The boxplots showed the enrichment scores of **(A)** immune infiltrations and **(B)** immune functions differed in the clusters. Boxplots depicted the differences in enrichment scores of metabolism-associated processes between glycosylation clusters, which included **(C)** amino acid metabolism, **(D)** carbohydrates metabolism, **(E)** lipid metabolism, and **(F)** other metabolism.

To further validate the glycosylation modification patterns and the potential biological behavior, GRGs identified by differentially expressed analysis and WGCNA were further inputted into the subsequent investigation. Primarily, 1945 DEGs between glycosylation subtypes including 788 up-regulated genes and 1157 down-regulated genes were visualized in the volcano plot (Figure 3A), and the top 100 ones are displayed in Figure 3B. Then 18 gene modules (Figure 3C) were recognized by WGCNA, and six modules (light yellow, brown, grey60, dark orange, dark grey, and tan) were closely related to glycosylation (Figure 3D). Eventually, 971 interactions (Figure 3E), considered GRGs (Supplementary Table S1), were uploaded into the consensus clustering analysis. Consistent with the subtype grouping of glycosylation modification patterns, the TCGA-LGGs cohort was divided into two distinct clusters, which were defined as glycosylation clusters A and B (Figure 3F). Also, the t-SNE algorithm data confirmed the rationality of the cluster assignments (Figure 3G). As expected, we found that patients in cluster A exhibited significantly shortened the survival time (Figure 3H; log-rank test). This demonstrated that two distinct glycosylation modification patterns did exist and work in LGGs. We further probed into the metabolism and immune microenvironment characteristics of the glycosylation clusters. In short, glycosylation cluster A showed higher scores of immune infiltrating cells (Figure 4A) and immune function (Figure 4B), except for CD8^+^ T cells, mast cells, and natural killer (NK) cells. Moreover, metabolic characteristics also showed significant differences (Figures 4B–E) between glycosylation clusters. Excitedly, these results were much similar to that of between glycosylation subtypes. Collectively, the concordance between prognostic, metabolic, and immune microenvironment characteristics in glycosylation clusters and subtypes confirmed again that glycosylation modification played a vital role in metabolic reprogramming and immune regulation, and this classification was steady and reliable.

### Development of the glycosylation scoring system as an independent prognostic factor for LGGs

**FIGURE 5:**
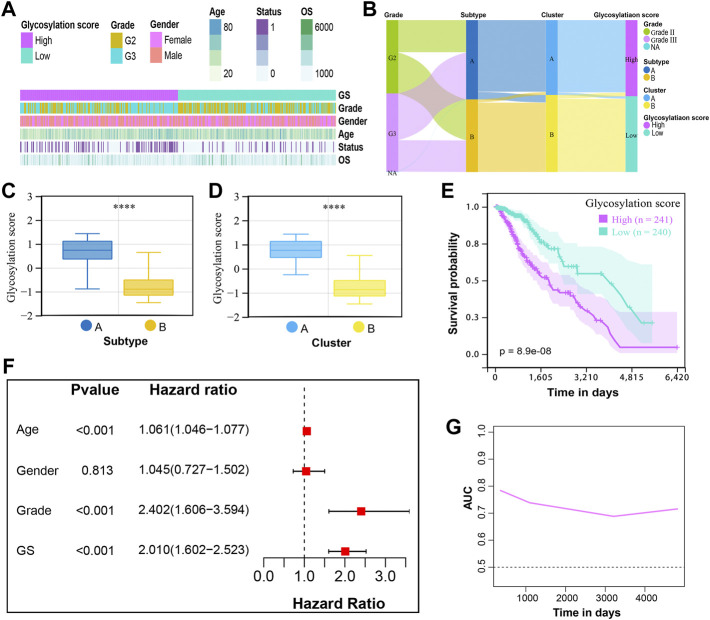
Development of the glycosylation scoring system and its prognostic values for LGGs. **(A)** Clinical features in the high and low glycosylation score subgroups. **(B)** Alluvial diagram of glycosylation score in groups with different grades, subtypes, and clusters. The boxplot showed the distribution of glycosylation score in the different **(C)** subtypes and **(D)** clusters. The *p*-values were both less than 0.0001. **(E)** K-M curves for patients with low and high glycosylation scores. **(F)** Multivariate Cox regression analysis for the glycosylation score and clinical variables. GS, glycosylation score. **(G)** Time-dependent AUC value of the glycosylation score demonstrated it as a robust and reliable prognostic index.

Although our findings revealed the role of glycosylation modification in prognosis, metabolic reprogramming, and immune modulation, these analyses could not accurately quantify the glycosylation modification patterns of individual tumors. Therefore, to enable the quantification of glycosylation patterns in individual LGGs patients, we developed a glycosylation scoring system termed as the glycosylation score. Based on the median glycosylation score of 0.009856, patients were classified into the high and low glycosylation score subgroups. Figure 5A showed the distribution of clinical traits including age, gender, grade, overall survival, and survival status between high and low glycosylation score subgroups, while Figure 5B further displayed the grade distribution, glycosylation subtypes, and clusters assignment of patients from distinct glycosylation score subgroups, which are summarized in Supplementary Table S2. The Wilcoxon test demonstrated that patients in subtype A had a higher glycosylation score compared to subtype B (Figure 5C); meanwhile, cluster A showed a higher glycosylation score than that of cluster B (Figure 5D). This indicated that a high glycosylation score could be closely related to poor prognosis, whereas a low glycosylation score could be closely related to a favorable prognosis. Of course, this hypothesis was confirmed by a subsequent survival analysis (Figure 5E) based on glycosylation scores. We analyzed the prognostic values of glycosylation scores in depth. The multivariate cox regression analysis demonstrated that the glycosylation score was an independent risk factor with its HR = 2.010 for LGGs (Figure 5F). Meanwhile, the time-dependent area under the curve (AUC) suggested that the glycosylation score had a robust and reliable value in predicting the prognosis for LGGs in the TCGA cohort (Figure 5G).

We further verified the predictive effectiveness of glycosylation score in three independent external cohorts according to the same glycosylation gene signatures and cutoff score. Consistent with findings in the TCGA cohort, patients with a high glycosylation score showed a significantly shortened survival than those with a low glycosylation score in validation cohorts including CGGA-mRNAseq_693 (Supplementary Figure S2A), CGGA-mRNAseq_325 (Supplementary Figure S2B), and CGGA-mRNA-array_301 (Supplementary Figure S2C). Similarly, superior time-dependent AUCs were observed in those cohorts (Supplementary Figures S2D–F).

### Glycosylation score is associated with metabolism and immune microenvironment features of LGGs

**FIGURE 6:**
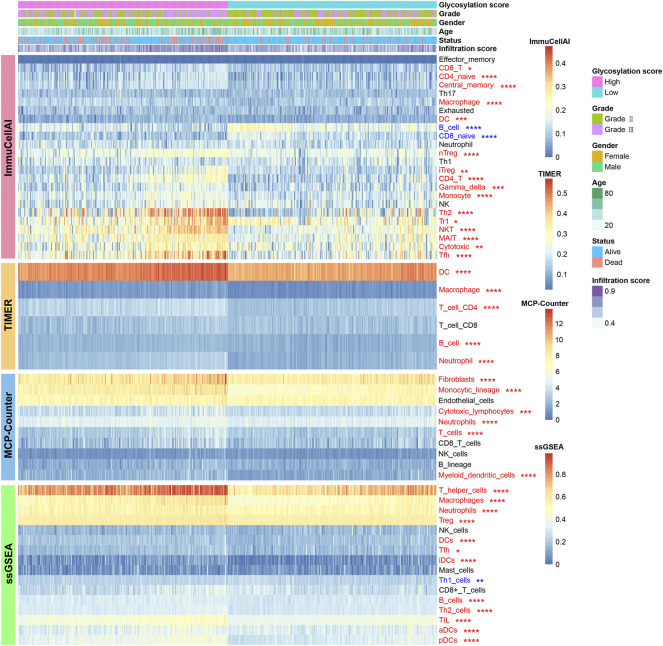
Landscape of infiltrating cells in the high and low glycosylation score subgroups. The heat map depicted the immune and stromal cell infiltration scores. Red represents cells infiltrated higher in the high glycosylation score subgroup and blue represents cells infiltrated lower in the high glycosylation score subgroup. *p < 0.05; **p < 0.01; ***p < 0.001; ****p < 0.0001.

**FIGURE 7:**
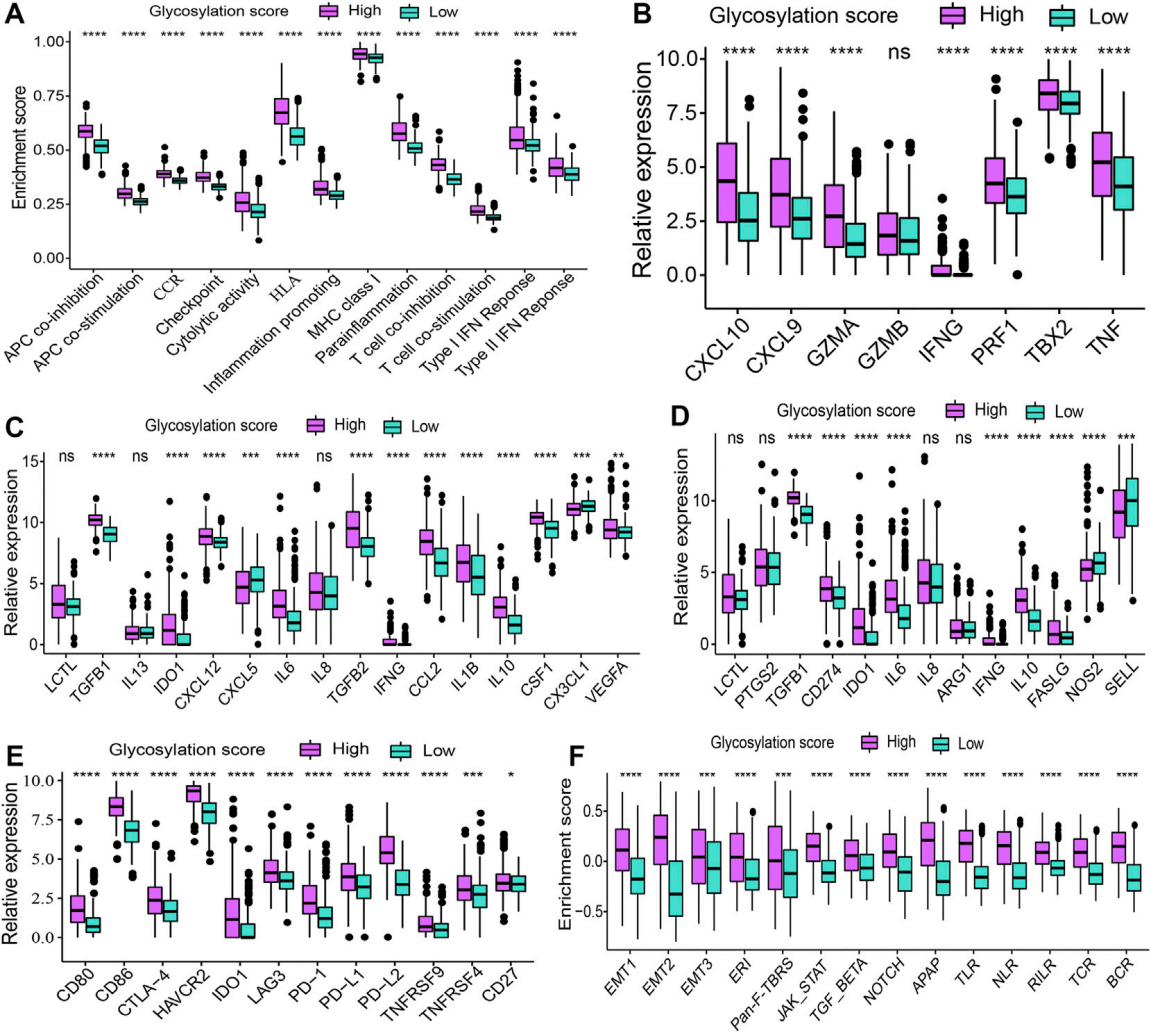
Cytokine, chemokine, and other related biological processes in glycosylation score subgroups. (*p < 0.05; **p < 0.01; ***p < 0.001; ****p < 0.0001; ns, not significant) **(A)** Difference in the enrichment score of immune functions between glycosylation score subgroups. **(B)** Difference in the gene expression related to immune activation between glycosylation score subgroups. The difference in the expression of **(C)** immunosuppressive cell recruitment factors and **(D)** immunosuppressive factors between glycosylation score subgroups. **(E)** Difference in the gene expression related to immune checkpoints between glycosylation score subgroups. **(F)** Difference in the gene expression of other related biological processes including immune activation, stromal activation, and cancer promotion between glycosylation score subgroups. ERI, extracellular matrix (ECM) receptor interaction; Pan-F-TBRS, pan-fibroblast TGFb response signature; APAP, antigen processing and presentation; TLR, Toll-like receptor; NLR, NOD-like receptor; RILR, RIG-I-like receptor; TCR, T cell receptor; BCR, B cell receptor.

The correlations between the glycosylation score and metabolism as well as immune microenvironment features were further explored in this study. Consistent with findings observed in distinct glycosylation patterns, the glycosylation score was strongly linked to metabolism activity (Supplementary Figure S3A) and immune microenvironment infiltrations (Figure 6). The results showed that the high glycosylation score subgroup was associated with enhanced activity in most amino acid metabolism (Supplementary Figure S3B) and carbohydrates metabolism (Supplementary Figure S3C); on the contrary, the low glycosylation score subgroup was associated with enhanced activity in most lipid metabolism (Supplementary Figure S3D) and other metabolism (Supplementary Figure S3E). Meanwhile, the distribution of infiltrating cells estimated by ImmuCellAI, TIMER, MCP-counter, and ssGSEA algorithms between the high and low glycosylation subgroups were also investigated. Our results (Figure 6) suggested that the high glycosylation score subgroup had a significantly higher infiltrating level than the low glycosylation score subgroup. Most of the infiltrating immune and stromal cells increased in the high glycosylation score subgroup, whereas T helper 1 (Th1) and CD8^+^ naïve cells decreased in this subgroup. Furthermore, there were higher scores of 13 immune functions in the high glycosylation score subgroup (Figure 7A), compared to the low glycosylation score subgroup. Taken together, the glycosylation score presented a close association with metabolism and immune microenvironment features of LGGs.

### Correlation of cytokine, chemokine, and other related biological processes in glycosylation score subgroups

To explore potential mechanisms correlated with different glycosylation score subgroups in depth, we compared the expression of chemokine and cytokine between subgroups. These cytokine and chemokine retrieved from published literature, of which, includes the transcripts correlated with immune activation (Figure 7B), immunosuppressive cell recruitment factors (Figure 7C), immunosuppressive factors (Figure 7D), and immune checkpoints (Figure 7E). We observed that most of these genes were expressed higher in the high glycosylation score subgroups, revealing the coexistence of a chronic inflammation state and a suppressed immune microenvironment in this subgroup. To better depict the role of glycosylation scores, we evaluated other related biological processes, including immune activation, stromal activation, and cancer promotion, in patients with LGGs. Consistent with the aforementioned observations, patients in the high glycosylation score subgroup exhibited a much higher enrichment score of immune activation–related biological processes (Figure 7F). On the other hand, higher stromal activation and cancer promotion scores were also enriched in this subgroup. Collectively, the glycosylation score did play a non-negligible role in affecting LGG progression.

### Potential small molecule compounds and drug sensitivity prediction

**FIGURE 8:**
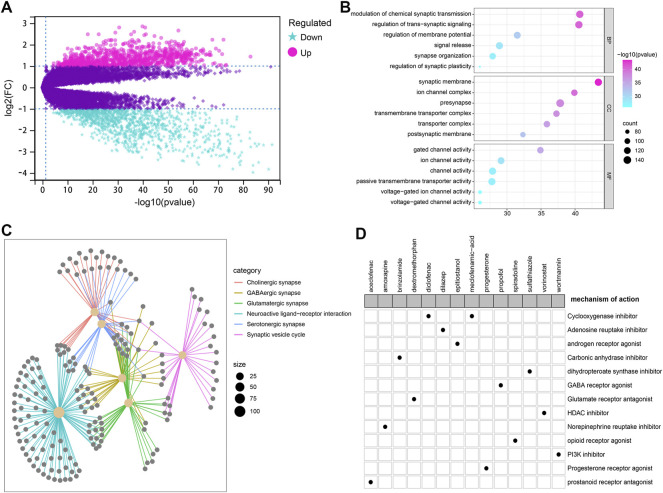
Potential small molecule compounds based on the glycosylation score. **(A)** Volcano plot showed 2090 DEGs between the high and low glycosylation score subgroups including 923 up- (red) and 1167 down-regulated (blue) genes. **(B)** Results of GO functional annotation for the DEGs, including biological process (BP), cellular component (CC), and molecular function (MF). **(C)** KEGG pathway enrichment analysis results showed the top six pathways ranked by the *p*-value. **(D)** CMap MoA analysis identified 13 mechanisms of action shared by 14 small therapeutic drugs potentially applicable for high glycosylation score patients.

To explore potential small therapeutic drugs for patients with high glycosylation scores, 2090 DEGs, including 923 up-regulated genes and 1167 down-regulated genes, between the high and low glycosylation score subgroups were identified (Figure 8A). The GO analysis results illustrated that the DEGs are primarily involved in modulation of chemical synaptic transmission, regulation of trans-synaptic signaling, synaptic membrane, ion channel complex, gated channel activity, and ion channel activity (Figure 8B). Meantime, these DEGs were significantly enriched in the cholinergic synapse, GABAergic synapse, glutamatergic synapse, neuroactive ligand–receptor interaction, serotonergic synapse, and synaptic vesicle cycle signaling pathways (Figure 8C). Based on the CMap MoA analysis, a total of 14 potential small therapeutic drugs (such as spiradoline, propofol, and dextromethorphan) and 13 drug mechanisms (such as opioid receptor agonist, GABA receptor agonist, and glutamate receptor antagonist) were identified (Figure 8D).

To explore the potential role of glycosylation score in the drug sensitivity prediction, we compared the IC50 value of 138 drugs in TCGA-LGGs patients by using the pRRophetic algorithm; of which, the results showed that there were 29 drugs (such as gefitinib, nilotinib, and axitinib) in which the estimated IC50 value were significantly lower in the low glycosylation score subgroup compared to the high glycosylation score subgroup (Supplementary Figure S4A), indicating that patients in the low glycosylation score subgroup might respond better to these chemotherapy drugs. Similarly, patients in the low glycosylation score subgroup had a higher IPS score, suggesting that patients in this subgroup might be more sensitive to immunotherapy (Supplementary Figure S4B).

## Discussion

Increasing evidence demonstrated that glycosylation plays an indispensable role in metabolism, immune response, and malignancy (Fujita et al., 2021; Hu et al., 2021; Pijnenborg et al., 2021), which is a complex and multistep process through multiple glycosyltransferase enzymes (Schjoldager et al., 2020). However, most studies concentrated on a single glycosyltransferase enzyme, and the overall metabolism and immune microenvironment landscape characteristics mediated by integrated glycosyltransferase enzymes have not been fully recognized. Therefore, the identification of distinct glycosylation modification patterns in the metabolism and immune microenvironment landscape characteristics will contribute to improve our understanding of the roles of glycosylation modification patterns and provide novel insights for making more effective managements of LGGs.

In the present study, we identified two distinct glycosylation modification patterns, characterized by different prognosis, metabolism, and immune microenvironment features. The glycosylation subtype A and cluster A were characterized by the higher carbohydrates and amino acid metabolism activity, higher level of infiltrating cells, and poor prognosis. However, an opposite modification pattern was observed in glycosylation subtype B and cluster B, that is, characterized by the higher lipid and other metabolism activity, lower level of infiltrating cells, and better prognosis. A previous study demonstrated that the tumor microenvironment plays a crucial role in immune response and tumor progression (Galon and Bruni, 2019). Appropriate stromal status contributes to migration of T cells, whereas loose or dense stromal might hinder the migration of T cells and limit their entry into the tumor parenchyma (Salmon et al., 2012). In addition, the abundance of immune cells would prevent them from penetrating tumor cell nests and being retained in the surrounding stroma (Joyce and Fearon, 2015). Consist with these findings, the glycosylation subtype A and cluster A infiltrated higher immune cells but with poor prognosis, reflecting an inefficient antitumor immune. Metabolic reprogramming is one of the well-established hallmarks of cancers, a flexible metabolic reorganization, which is tailored to meet their energy requirement and maintain the homeostasis of environmental conditions (An and Duan, 2022; Corchado-Cobos et al., 2022). These metabolic variations include the synthesis of protein, cell membranes, and nucleic acids, which all promote cell proliferation (Corchado-Cobos et al., 2022). Confusingly, lipid metabolism is widely believed to be oncogenic and facilitate tumor progression; our analysis showed that hyperactive lipid metabolism was related to a better prognosis, indicating an anticancer role of lipid metabolism in LGGs. However, our finding was confirmed by present studies (Peng et al., 2018; Shen et al., 2020). This is an attractive and novel theory, and the underlying mechanism is still ambiguous and needs to be confirmed.

Furthermore, we developed a scoring system termed ‘glycosylation score’ by using GSVA to quantify the glycosylation modification patterns and provide more precise management strategies for individual LGGs patients. GSVA, a non-parametric and unsupervised method, can map gene expression data from a sample into predefined gene sets and summarize it into a single enrichment score for each gene set. A significant benefit of this gene set–based method is the rationality that genes functioned collectively and varied by genetic modifications or disease states. Another advantage is the comparability that GSVA could calculate an expression-level statistic with different dynamic ranges to a common scale. In other words, the enrichment scores of the same gene set in different datasets are comparable (Hänzelmann et al., 2013). While, many existing studies adopted least absolute shrinkage and selection operator (LASSO) regression (Tan et al., 2022; Yang and Zhang, 2022) or principal component analysis (PCA) (Zhang et al., 2020; Chong et al., 2021), which cannot satisfy the aforementioned two advantages at the same time. In result, the glycosylation modification pattern subtype A and cluster A showed a higher glycosylation score, while the glycosylation modification pattern subtype B and cluster B exhibited a lower glycosylation score. Consistent with the aforementioned findings, the glycosylation score was closely related to prognosis, metabolism status, and immune microenvironment cell-infiltrating. Moreover, integrated analyses also demonstrated that the glycosylation score was an independent prognostic index in LGGs, and its predictive efficacy was evaluated by a time-dependent ROC analysis and validated in three independent external cohorts. Collectively, the glycosylation score did play a non-negligible role in LGGs progression and became a novel and reliable tool for quantifying glycosylation modification patterns in LGGs.

Immunotherapy, an emerging potential therapy for cancers, has attracted considerable attention in recent years. However, only a subset of patients responds well to this therapy (Ott et al., 2021). Thus, it is particularly important to stratify LGGs patients and screen the ones who may benefit from immunotherapy. Further analyses highlighted that the high glycosylation score subgroup is closer to a microenvironment characterized by chronic inflammation, immunosuppression, and tumor promotion. We observed that this subgroup showed higher levels of cytokine, chemokine, and biological processes activity, involving immune activation, immunosuppression, stromal activation, and cancer promotion. Previous studies demonstrated that activation of EMT- and TGF-β–related pathways could lead to a decrease of lymphocyte cells into tumor parenchyma (Galon and Bruni, 2019) and a weakness of their cytotoxicity effects (Salmon et al., 2012), which were considered T-cell suppressive. In addition, the previous publication confirmed that stromal activation represented a major mechanism of immune evasion (Galon and Bruni, 2019) and mediated resistance to checkpoint immunotherapy (Mariathasan et al., 2018). Accordingly, we speculated that the patients with high glycosylation scores are more likely to experience resistance to immunotherapy. Next, the findings of higher IPS distributed in the low glycosylation score subgroup also suggested that patients with low glycosylation scores may respond better to immunotherapy.

To further gain novel insight into the applicability of glycosylation score and provide clues for optimizing the personalized treatment of patients, the CMap database was applied to explore potential drugs and corresponding targets for high glycosylation score subgroup patients. A total of 14 potential small therapeutic drugs and 13 drug mechanisms were identified, which could contribute to providing new treatment opportunities for LGGs patients; therefore, these findings are needed to be validated by further experiments. Furthermore, the drug sensitivity analysis by the pRRophetic algorithm revealed that 29 antitumor drugs such as gefitinib, nilotinib, and axitinib responded distinctly differently between high and low glycosylation score subgroups. These results suggested that the glycosylation score influenced the therapeutic efficacy of chemotherapy and targeted therapy. Therefore, this points out new directions for the potential clinical application of the glycosylation score. A comprehensive strategy combing immunotherapy, chemotherapy, and targeted therapy based on glycosylation score may be excellent.

In summary, the glycosylation score could be applied to comprehensively evaluate the glycosylation modification patterns, along with their corresponding immune microenvironment and metabolism features. Furthermore, the glycosylation score could be used as an independent prognostic index for predicting LGGs patients’ survival. More importantly, our results also provided a novel insight into promoting personalized therapy in the future, which may contribute to developing novel therapeutic drugs or exploring promising drug combination therapy strategies.

## Data Availability

The datasets presented in this study can be found in online repositories. The names of the repository/repositories and accession number(s) can be found in the article/Supplementary Material.
