# Supplementary figures and images for: Glycosylation modification patterns reveal distinct tumor metabolism and immune microenvironment landscape in lower-grade gliomas
Source: Front Cell Dev Biol. 2022 Aug 25;10:886989. doi: 10.3389/fcell.2022.886989 (PMC9452883; doi:10.3389/fcell.2022.886989)

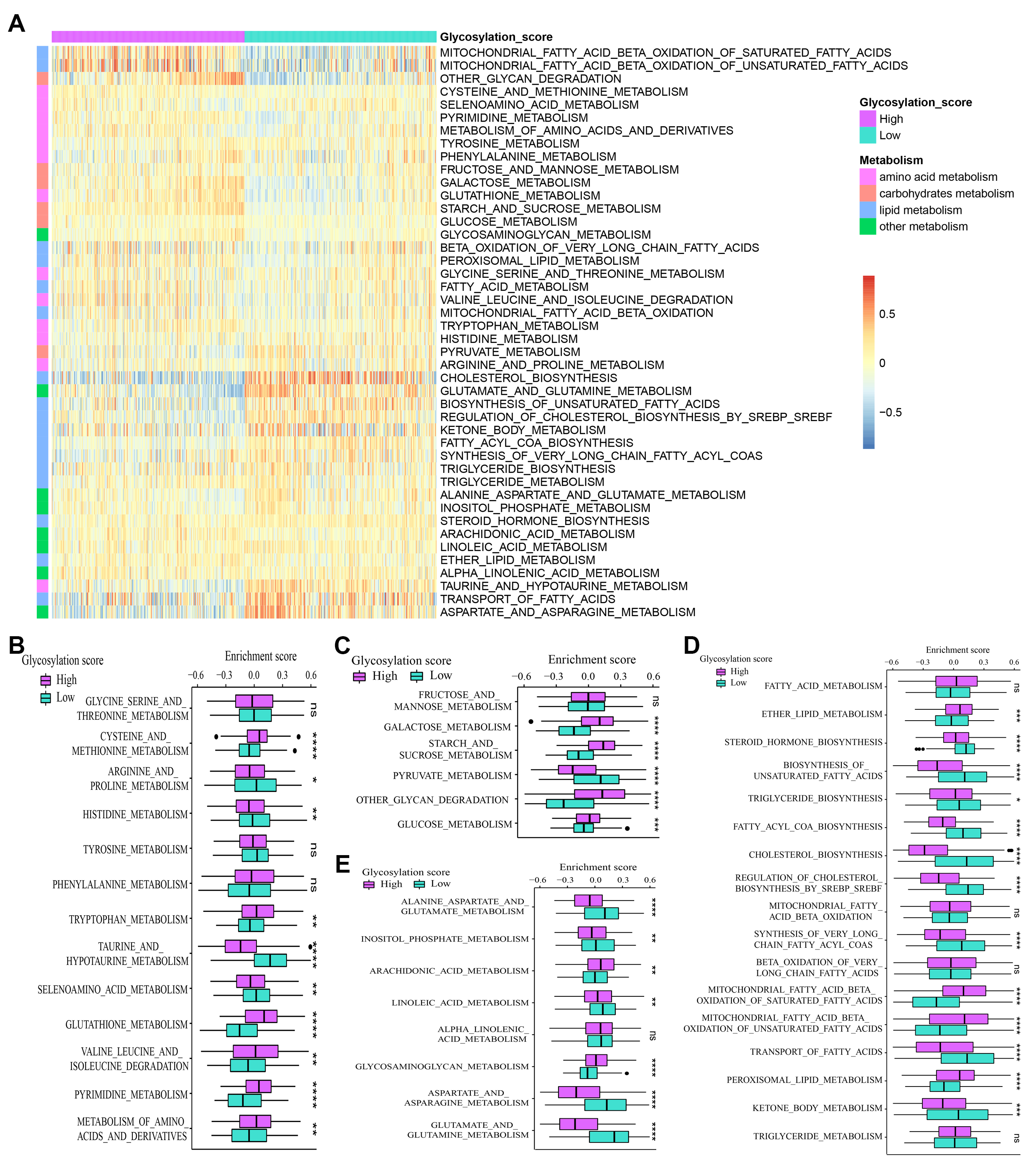

Supplement: Supplementary file 1 [file Image3.TIF]

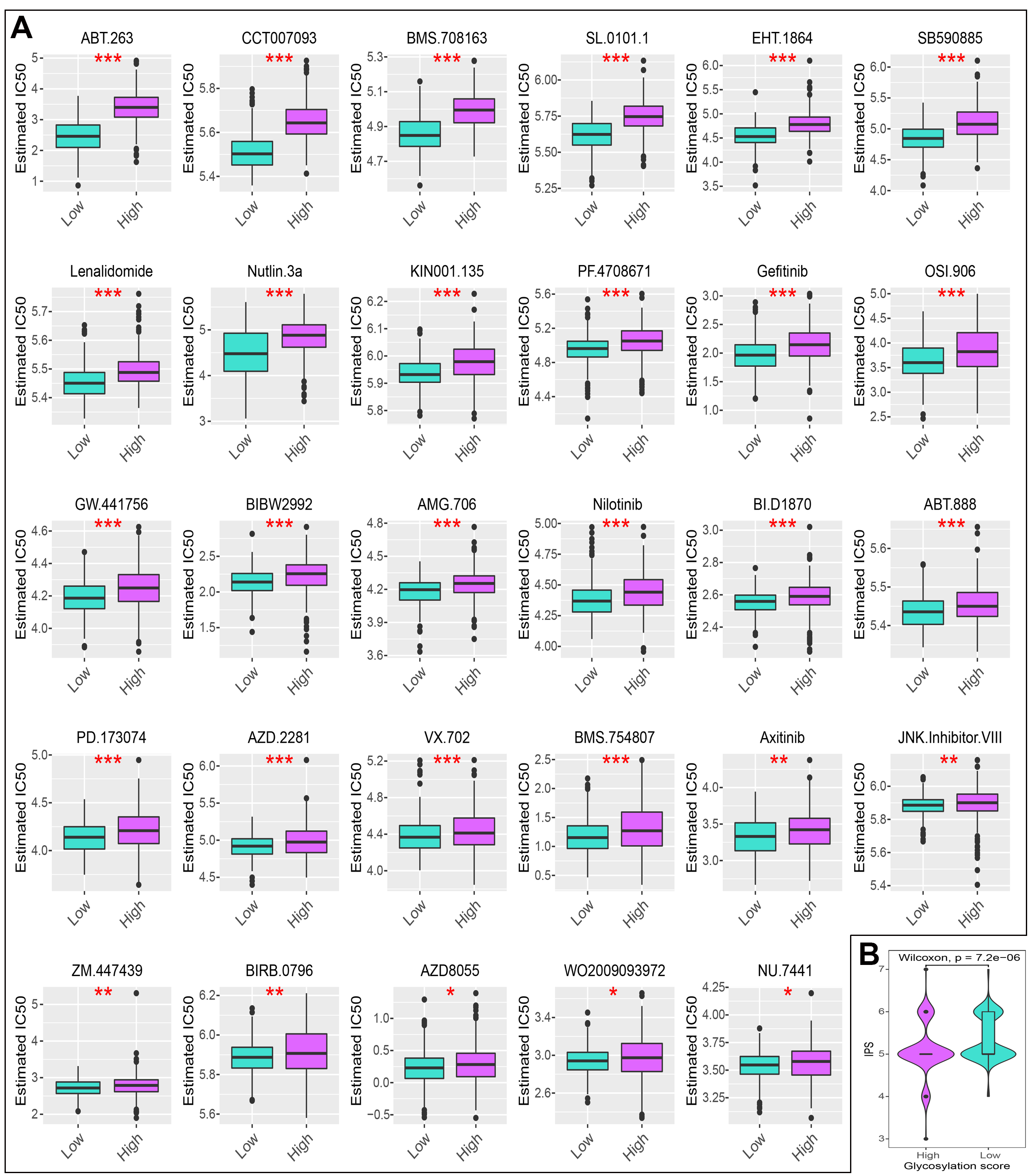

Supplement: Supplementary file 2 [file Image4.TIF]

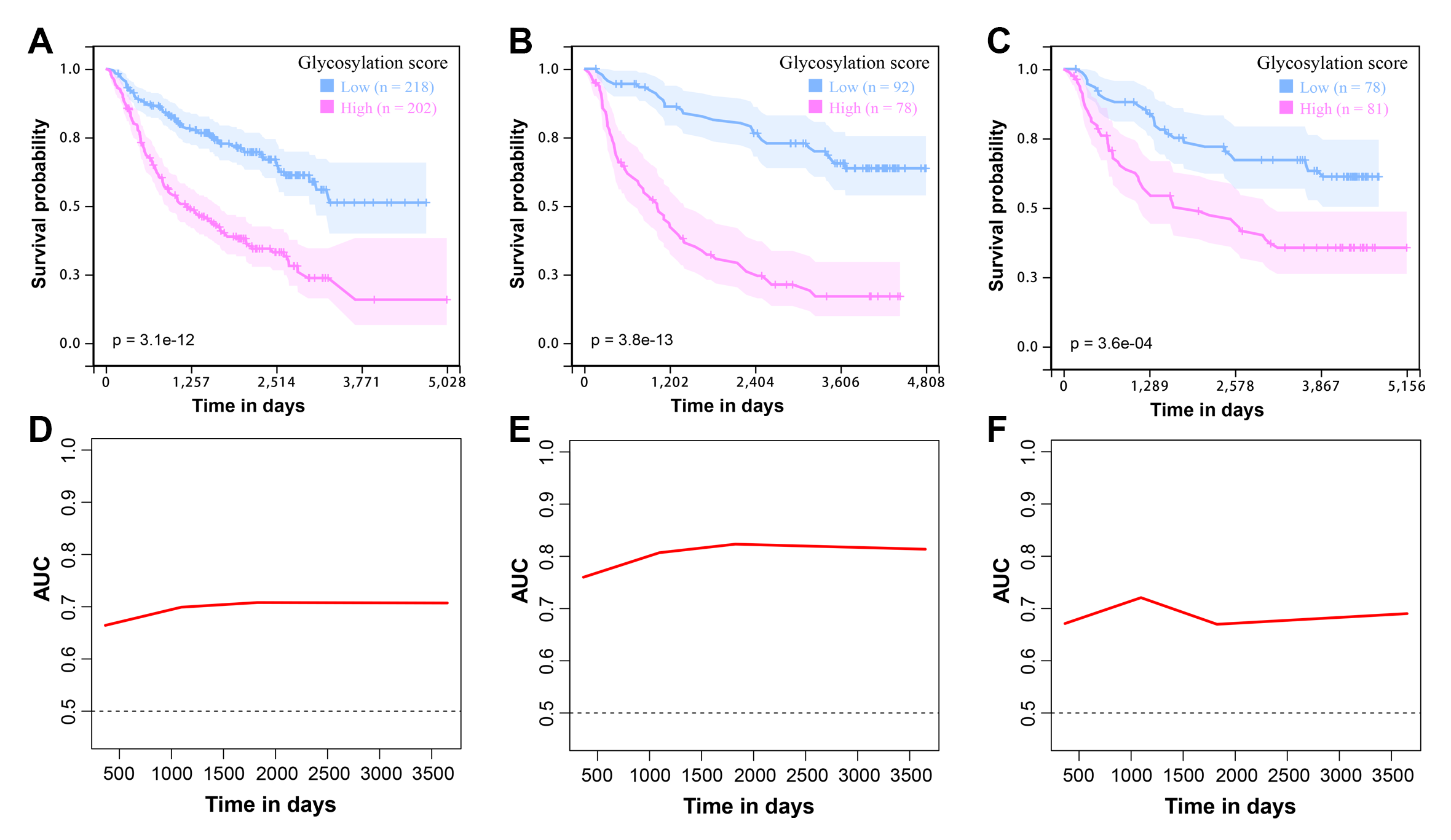

Supplement: Supplementary file 3 [file Image2.TIF]

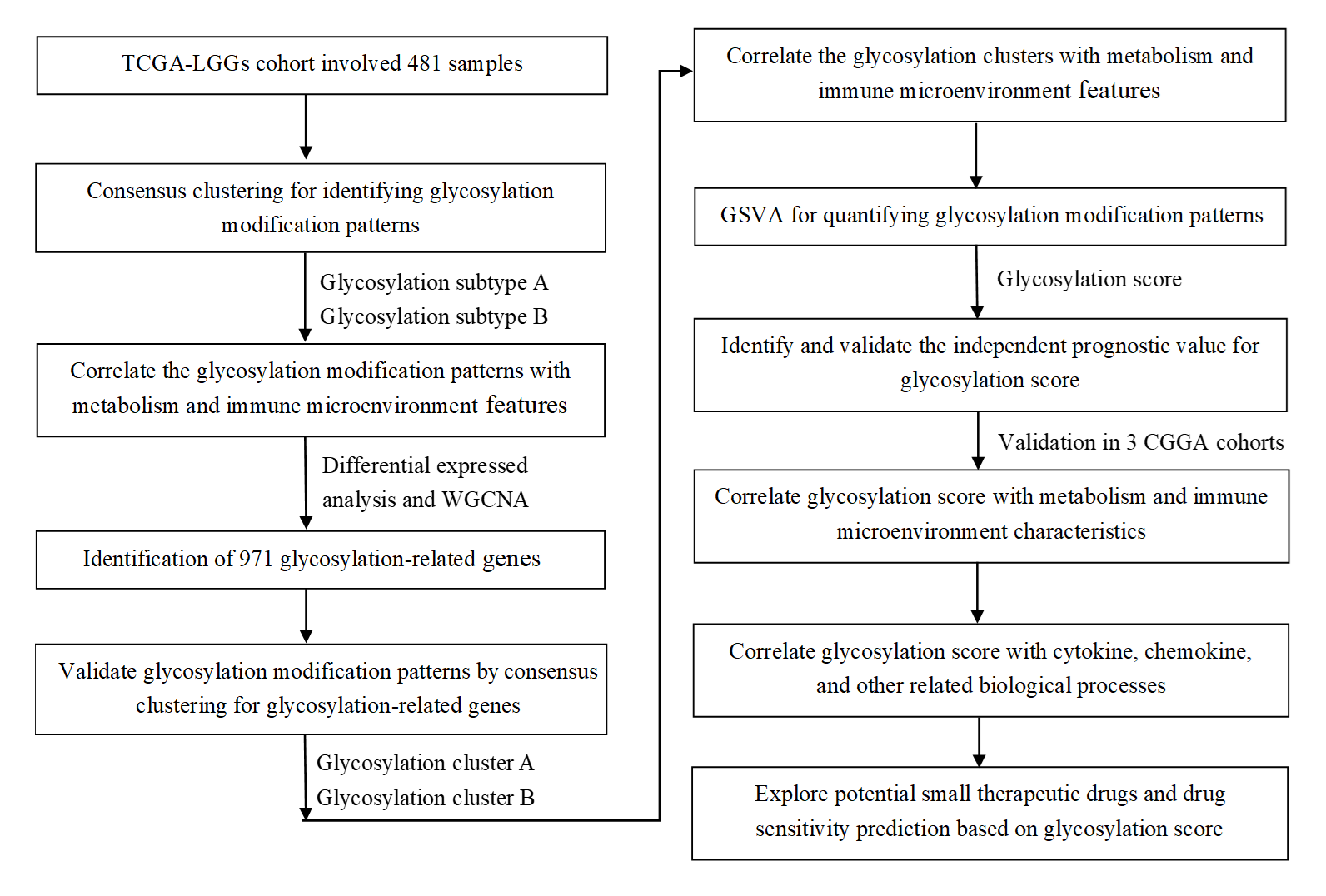

Supplement: Supplementary file 4 [file Image1.TIF]
